# Injecting OVD temporarily seal the edge of the retinectomy to prevent subretinal perfluorocarbon liquid droplets retention and retinal slippage during fluid-air exchange

**DOI:** 10.1186/s40942-026-00828-z

**Published:** 2026-03-09

**Authors:** Zifang He, Yinong Guo, Yilinuer Yilihaer, Suyu Liu, Yifei Tang, Lv Xie, Ye Zhang, Yajun Liu, Zhenggao Xie

**Affiliations:** 1 Department of Ophthalmology, Nanjing Drum Tower Hospital, The Affiliated Hospital of Medical School, Nanjing University, Nanjing, 210008 China; 2 Department of Ophthalmology, Nanjing Drum Tower Hospital Clinical College of Nanjing Medical University, Nanjing, China; 3 Department of Ophthalmology, Dongtai Traditional Chinese Medicine Hospital, Yancheng City, Jiangsu Province China

**Keywords:** Retinectomy, Retinal slippage, Vitrectomy, Perfluorocarbon liquid retention

## Abstract

**Background:**

To investigate effect of the injection of ophthalmic viscosurgical device (DisCoVisc) temporarily seal the edge of the retinectomy to prevent subretinal perfluorocarbon liquid (PFCL) droplets retention and retinal slippage during fluid-air exchange.

**Methods:**

PFCL was injected, with the fluid level extended 3–5 mm above the posterior edge of the retinectomy. Laser photocoagulation was applied around the edge of the retinectomy. DisCoVisc was then injected under PFCL and evenly applied along the edge of the retinectomy to achieve temporary and complete sealing. Under infusion fluid, PFCL was completely removed. Then fluid-air exchange was applied. Finally, silicone oil was injected for intravitreal tamponade.

**Results:**

A total of 9 patients (9 eyes) were included in this study, all of whom underwent retinectomy. On postoperative day 1, the retinas of all patients remained fully attached without any flap inversion or slippage. No subretinal PFCL droplets were detected. At last visit, the mean IOP was 15.61 ± 7.18 mmHg, without significant difference compared to preoperative IOP (13.39 ± 4.97 mmHg) (*P* = 0.455), but the mean post-operative BCVA (1.01 ± 0.62) logMAR (Snellen equivalent 20/200) with a significant difference compared to preoperative BCVA (2.04 ± 1.42) logMAR (Snellen equivalent 20/2190) (*P* = 0.031).

**Conclusions:**

Injecting DisCoVisc under PFCL to temporarily seal the edge of the retinectomy effectively prevents infusion fluid and PFCL droplets from entering the subretinal space during fluid–air exchange, thereby reducing the risk of retinal slippage and subretinal PFCL retention.

**Clinical trial number:**

Not applicable.

**Supplementary Information:**

The online version contains supplementary material available at 10.1186/s40942-026-00828-z.

## Background

Proliferative vitreoretinopathy (PVR) is the primary cause of redetachment following initially successful surgery for rhegmatogenous retinal detachment (RRD). Multiple factors such as ocular trauma, giant retinal tears, and chronic retinal detachment have been associated with an increased risk of PVR development [1]. While complete pars plana vitrectomy (PPV) with thorough membrane peeling is essential for effective PVR management, residual traction or retinal shortening may lead to surgical failure. To achieve anatomical success, relaxing retinectomy is often required [2]. One of the major challenges in retinectomy surgery lies in the tendency of the posterior flap of the retinal tear to slip posteriorly toward the macula during fluid–air exchange. Another is the entry of small droplets of perfluorocarbon liquid (PFCL) into the subretinal space, resulting in subretinal or subfoveal PFCL droplets retention [3]. To prevent retinal slippage, some surgeons have attempted the “head-tilt drainage” technique to completely evacuate intraocular PFCL and infusion fluid [4]. However, in clinical application, less experienced surgeons may still be unable to avoid retinal slippage and subretinal PFCL retention. Others have employed silicone oil–PFCL exchange to stabilize the retina and avoid slippage [5]. In our one previous study, we injected the dispersive ophthalmic viscosurgical device (OVD, DisCoVisc) under infusion fluid onto the inverted internal limiting membrane (ILM) flap. The adhesive property of DisCoVisc helps stabilize the flap on the surface of the macular hole [6]. In another study, we found that DisCoVisc could adhere to and seal the retinal breaks to prevent detached retina elevation, substituting for PFCL in vitrectomy for rhegmatogenous retinal detachment [7]. Additionally, Ruparelia et al. reported the use of viscoelastic injection under PFCL to stabilize ILM flaps [8]. Based on these two methods, we developed a novel technique wherein DisCoVisc is injected onto the edge of the posterior flap of retinectomy to temporarily seal the edge of tear. This method aims to prevent the entry of infusion fluid and PFCL droplets into the subretinal space during fluid-air exchange, thereby reducing the risk of retinal slippage and subretinal PFCL droplets retention.

## Methods

This was a case series study. All patients were informed of the surgical plan and potential risks prior to surgery and provided written informed consent. All patients have provided informed consent for publication of the case. The study was approved by the Ethics Committee of Nanjing Drum Tower Hospital, the Affiliated Hospital of Medical School, Nanjing University. Inclusion criteria: Retinectomy involving 90 degrees of arc or more. Exclusion criteria: Retinectomy involving less than 90 degrees of arc.

### Surgical technique

**Fig. 1:**
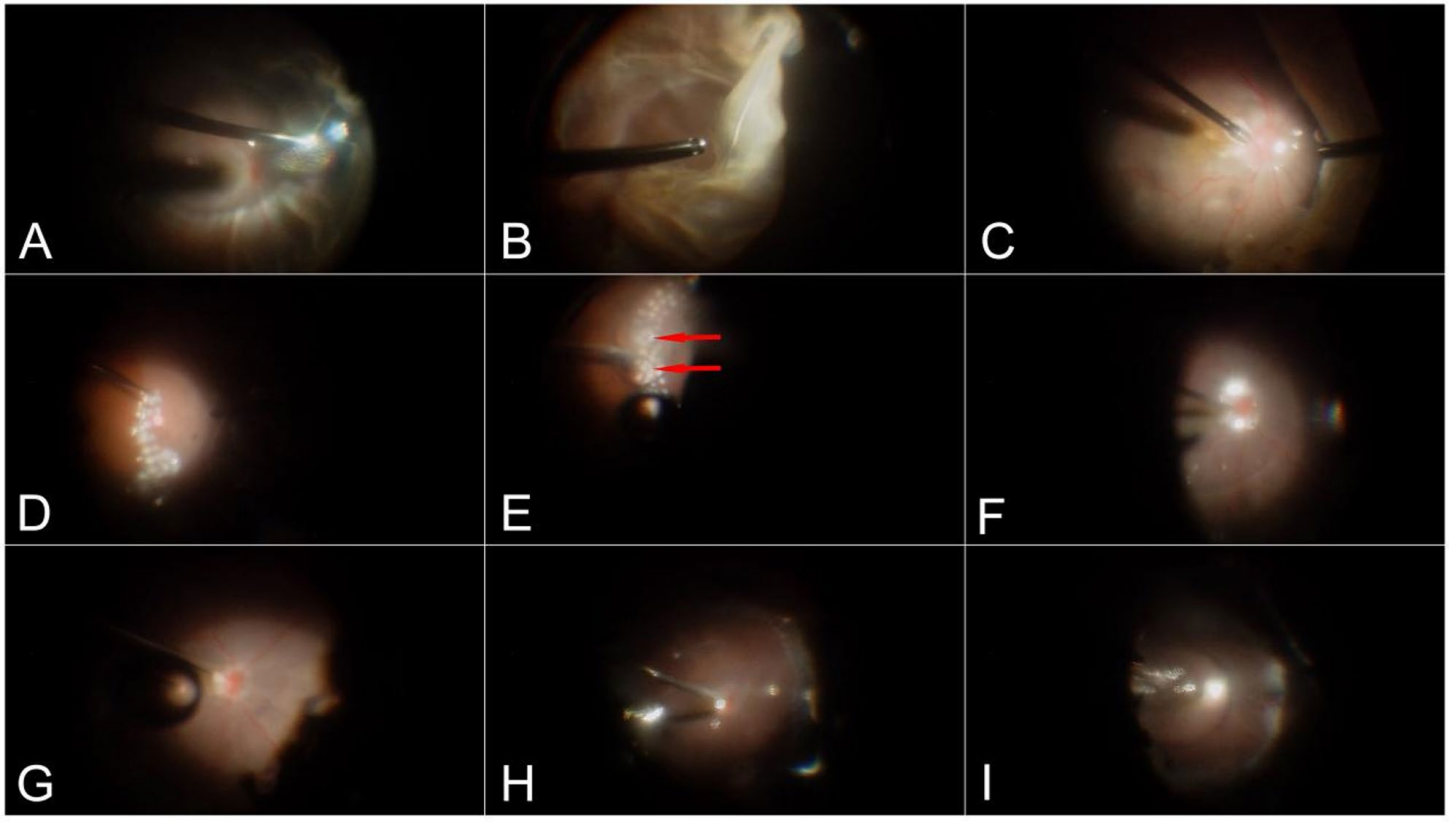
Procedures of sealing the edge of retinotomy using DisCoVisc under PFC. **A**: The patient presents with funnel retinal detachment and anterior PVR. **B**: A circumferential retinotomy is performed on the peripheral retina. **C**: Slow injection of PFCL into the vitreous cavity to flatten the retina. **D**: Laser photocoagulation (3–4 rows) is applied around the retinal edge. **E**: DisCoVisc is injected along the edge, half over the neurosensory retina and half over the retinal pigmental epithelium (red arrows), to seal the entire circumference of the retinectomy. **F**: PFCL is slowly aspirated under infusion fluid. **G**: After PFCL removal, no posterior slippage of the retina is observed. **H**: The retina remains attached after fluid–air exchange with no retinal slippage or residual PFCL droplets. I: Silicone oil is injected into the vitreous cavity

A standard 23-gauge three-port pars plana vitrectomy was performed (Constellation, Alcon, Fort Worth, TX). Triamcinolone acetonide was used to stain the vitreous cortex on the retinal surface, which was then thoroughly removed using a retinal brush. The peripheral and basal vitreous were removed as thoroughly as possible to relieve vitreoretinal traction, followed by peripheral retinectomy. PFCL was injected, with the fluid level extended 3–5 mm above the posterior edge of the retinectomy. Under PFCL, laser photocoagulation (3–4 rows) was applied around the edge of the retinectomy. DisCoVisc (Alcon, Fort Worth, TX) was then injected under PFCL and evenly applied along the edge of the retinectomy to achieve temporary and complete sealing of the tear. Under infusion fluid, PFCL was completely removed using a vitreous cutter or flute needle. This was followed by fluid–air exchange to remove the remaining infusion fluid. At this stage, the retina was fully reattached. Finally, silicone oil was injected for intravitreal tamponade. (Fig. 1; see Video 1: Retinectomy, Supplemental Digital Content 1).

### Statistical analysis

Statistical analysis was performed using SPSS software version 23.0. Quantitative data were expressed as mean ± standard deviation (SD). Paired *t*-tests were used to compare intraocular pressure (IOP) and best-corrected visual acuity (BCVA) before and after surgery. A *P* value < 0.05 was considered statistically significant.

## Results

From April 2024 to June 2025, a total of 9 patients (9 eyes) were included in the study. All 9 eyes underwent retinectomy. Seven cases involved the right eyes and 2 cases involved left eyes. Nine patients were male (9 eyes). The mean age was 54.11 ± 14.13 years (range, 25 to 73 years). The mean preoperative IOP was 13.39 ± 4.97 mmHg (range, 5.6 to 23 mmHg), and the mean preoperative BCVA was 2.04 ± 1.42 logMAR (Snellen equivalent 20/2190) (range, 0.52 to 3.52). The extent of the retinectomy ranged from 120° to 360°, with a mean of 210.00 ± 96.04°. The mean intraoperative volume of DisCoVisc injected was 0.44 ± 0.13 mL (range, 0.3 to 0.6 mL). On postoperative day 1, the retinas of all patients remained fully attached without any flap inversion or slippage. No subretinal PFCL droplets were detected on fundus examination or wide-field optic coherence tomography (OCT). At last visit, the mean IOP was 15.61 ± 7.18 mmHg (range, 8.5 to 31.0 mmHg), without significant difference compared to preoperative IOP (*t* = -0.86, *P* = 0.455), but the mean post-operative BCVA (1.01 ± 0.62) logMAR (Snellen equivalent 20/200) with a significant difference compared to preoperative BCVA (*t =* 2.605, *P =* 0.031) (Table 1).

**Table 1:** Baseline demographic and clinical characteristics of the patients, and postoperative follow-up outcomes

Case No.	Gender (F/M) /Age (years)/Eye (*R*/L)	Extent of Retinectomy (degree)	DisCoVisc Volume (ml)	Intraoperative Retinal Slippage	Pre-op BCVA	Post-op BCVA	Pre-op IOP (mmHg)	Post-op IOP (mmHg)	Post-op Lens status	PFCL Droplets Retention (Y/*N*)	Post-op Retinal Slippage (Y/*N*)
					*t* = 2.605, *P* = 0.031		*t* =-0.860, *P* = 0.455				
1	M/42/L	180	0.4	N	20/80	20/50	15	15.2	Aphakia/Capsule (Y)	N	N
2	M/58/R	180	0.5	N	20/500	20/1,000	13	31	Aphakia/Capsule (Y)	N	N
3	M/57/L	360	0.6	N	HM/20 cm	20/125	12	9.8	Aphakia/Capsule (Y)	N	N
4	M/73/R	120	0.3	N	HM/20 cm	20/400	13	22	Aphakia/Capsule(N)	N	N
5	M/62/R	150	0.4	N	20/125	20/67	7.9	13	Aphakia/Capsule (N)	N	N
6	M/62/R	120	0.3	N	20/67	20/50	23	13	Aphakia/Capsule (Y)	N	N
7	M/47/R	150	0.3	N	HM/20 cm	CF/40 cm	5.6	10	Aphakia/Capsule (Y)	N	N
8	M/61/R	270	0.6	N	20/200	20/133	16	8.5	Aphakia/Capsule (N)	N	N
9	M/25/R	360	0.6	N	HM/20 cm	20/167	15	18	Aphakia/Capsule (Y)	N	N

**Notes**: F, female; M: male; R, right; L, left; BCVA, best-corrected visual acuity; IOP, intraocular pressure; Pre-op, preoperative; Post-op, post-operative; Y, yes; N, no; PFCL, perfluorocarbon liquid

## Discussion

This study reports the clinical outcomes of the injection of OVD (DisCoVisc) temporarily seal the edge of the retinectomy in nine consecutive patients with complex retinal detachment. At the final follow-up, successful retinal reattachment was achieved in all cases, with varying degrees of improvement in BCVA. No cases of retinal slippage or residual subretinal PFCL droplets were observed postoperatively. Elevated IOP was noted on postoperative day 1 in three patients, which was attributed to the use of DisCoVisc and was controlled with anti-glaucoma treatment. At the final follow-up, one patient exhibited increased IOP, possibly related to trabecular meshwork dysfunction following vitrectomy, which was also managed with topical anti-glaucoma therapy. No serious complications, such as infectious endophthalmitis, occurred.

Retinectomy is a well-established and effective vitreoretinal technique for treating retinal detachment in eyes with advanced PVR [9]. During retinectomy retinal slippage toward the posterior pole commonly occurs during fluid–air exchange, primarily attributable to the presence of residual subretinal fluid [10]. To ensure complete drainage of subretinal fluid and reduce the risk of retinal slippage, three main strategies are commonly employed in clinical practice: (1) Conventional fluid–air exchange method [11]: During fluid–air exchange, a flute needle is used to patiently and repeatedly aspirate residual infusion fluid and PFCL along the posterior edge of the retinectomy. Despite these steps, our clinical experience suggests that a small number of cases still exhibit residual PFCL droplets in the subretinal or subfoveal space. We performed an in vitro simulation experiment, which showed that under continuous infusion, PFCL bubbles appeared spherical due to the interfacial tension between PFCL and the infusion fluid. Once the overlying infusion fluid was removed, this interfacial tension disappeared and the PFCL bubbles flattened due to surface tension (see Video 2, Supplemental Digital Content 2). (2) Head-tilt drainage method [4]: PFCL is injected to a level slightly above the posterior edge of the retinectomy. One trocar is blocked with a plug. The patient’s head is tilted so that the retinectomy is in the most dependent position. The trocar is then slightly withdrawn to allow PFCL and infusion fluid to be expelled under the driving force of gas pressure. (3) Silicone oil–PFCL exchange method [5]: The infusion is stopped, and PFCL is injected to completely displace the infusion fluid. Silicone oil is then injected while simultaneously aspirating PFCL with a flute needle. All three methods require advanced surgical skills. Objectively, none can completely eliminate the risk of retinal slippage or subretinal entry of PFCL, particularly in less experienced hands.

Subretinal perfluorocarbon liquid (PFCL) retention is a relatively common complication following vitreoretinal surgery, particularly in cases of giant retinal tears and extensive retinal incisions, with an incidence ranging from 0.9% to 11.1% [12]. The underlying cause of subretinal PFCL may be related to excessively large tears or incisions that are difficult to close, facilitating the slippage of the PFCL droplets into the subretinal space. PFCL can persist either beneath the fovea or in extrafoveal locations, thereby interfering with retinal reattachment. Studies have shown that subfoveal PFCL retention can lead to decreased or even complete loss of vision. Furthermore, through mechanisms of mechanical compression and chemical toxicity, it can induce damage to the retinal pigment epithelium and photoreceptor cells [13–14].

Ophthalmic viscosurgical devices(OVDs) commonly used in clinical practice can be classified into two major categories: cohesive OVDs with high viscosity and dispersive OVDs with medium to low viscosity [15]. DisCoVisc is a novel single-agent formulation that combines the advantages of both cohesive and dispersive OVDs. Its components are 1.6% sodium hyaluronate and 4% chondroitin sulfate. Its high molecular weight (1700 kDa) endowing it with the static viscosity of cohesive OVDs. It also has a moderate cohesion/dispersion index (CDI) (CDI = 12), which makes it exhibit dispersion characteristics [16]. Unlike traditional cohesive OVDs such as sodium hyaluronate, DisCoVisc adheres firmly to intraocular tissue surfaces and is not easily flushed away by infusion fluid flow (see Video 3, Supplemental Digital Content 3). Following the approach reported by Ruparelia et al. [8], who injected viscoelastic under PFCL, we developed a technique in which DisCoVisc is applied under PFCL along the posterior edge of the retinectomy to temporarily seal the retinal edge. The function of DisCoVisc is similar to that of glue, temporarily adhering the edges of the retinal incision. This aims to prevent infusion fluid and PFCL droplets from entering the subretinal space during fluid–air exchange, thereby avoiding retinal slippage and subretinal PFCL droplets retention.

Benefits of this technique include: (1) It avoids the inconvenience of head tilting drainage method, especially in patients with cervical spine disorders. (2) It reduces the risk of PFCL droplets entering the subretinal space during direct fluid–air exchange. (3) By temporarily sealing the edge of a retinectomy with DisCoVisc and removing PFCL under infusion fluid, the PFCL bubble retains its spherical shape due to interfacial tension, with well-defined margins, making it highly visible and thus ensuring complete removal. (4) With sufficient PFCL injection, subretinal fluid can be thoroughly displaced. Once the retinectomy edge is completely sealed by DisCoVisc, the retina can fully reattach to the eyewall, minimizing the risk of posterior retinal slippage during fluid–air exchange.

Potential limitations of this technique include residual DisCoVisc in the eye may cause transient IOP elevation postoperatively, but this can be effectively managed with topical antiglaucoma medications. Additionally, DisCoVisc is not commercially available in certain regions. In such instances, alternative cohesive OVDs may be employed; however, clinical outcomes may be suboptimal compared to those achieved with DisCoVisc.

In conclusion, injecting DisCoVisc under PFCL to temporarily seal the edge of a retinectomy effectively prevents infusion fluid and PFCL droplets from entering the subretinal space during fluid–air exchange, thereby reducing the risk of retinal slippage and subretinal PFCL retention. 

## Supplementary Information

Below is the link to the electronic supplementary material.

Supplementary Material 1: Video. 1: DisCoVisc in Retinectomy.mp4.

Supplementary Material 2: Video. 2: In vitro simulation experiment-1.mp4.

Supplementary Material 3: Video. 3: In vitro simulation experiment-2.mp4.

## Data Availability

The datasets generated and/or analyzed during the current study are notpublicly available due to institutional data protection policies and patientprivacy regulations but are available from the corresponding author (ZG.X.) onreasonable request.

## Abbreviations

PFCL: Perfluorocarbon liquid
PVR: Proliferative vitreoretinopathy
RRD: Rhegmatogenous retinal detachment
PPV: Pars plana vitrectomy
ILM: Internal limiting membrane
SD: Standard deviation
IOP: Intraocular pressure
BCVA: Best-corrected visual acuity
OCT: Optic coherence tomography
OVDs: Ophthalmic viscosurgical devices
OVD: Ophthalmic viscosurgical device
